# Carbon Amendments Influence Composition and Functional Capacities of Indigenous Soil Microbiomes

**DOI:** 10.3389/fmolb.2019.00151

**Published:** 2020-01-09

**Authors:** José Pablo Dundore-Arias, Sarah C. Castle, Laura Felice, Ruth Dill-Macky, Linda L. Kinkel

**Affiliations:** ^1^Department of Biology and Chemistry, California State University, Monterey Bay, Seaside, CA, United States; ^2^Department of Plant Pathology, University of Minnesota, Saint Paul, MN, United States; ^3^Plant Science Research Unit, USDA-ARS, Saint Paul, MN, United States

**Keywords:** streptomyces, soil microbiome, carbon amendments, soil mesocosms, pathogen inhibition, suppressive soils

## Abstract

Soil nutrient amendments are recognized for their potential to improve microbial activity and biomass in the soil. However, the specific selective impacts of carbon amendments on indigenous microbiomes and their metabolic functions in agricultural soils remain poorly understood. We investigated the changes in soil chemical characteristics and phenotypes of *Streptomyces* communities following carbon amendments to soil. Mesocosms were established with soil from two field sites varying in soil organic matter content (low organic matter, LOM; high organic matter, HOM), that were amended at intervals over nine months with low or high dose solutions of glucose, fructose, malic acid, a mixture of these compounds, or water only (non-amended control). Significant shifts in soil chemical characteristics and antibiotic inhibitory capacities of indigenous *Streptomyces* were observed in response to carbon additions. All high dose carbon amendments consistently increased soil total carbon, while amendments with malic acid decreased soil pH. In LOM soils, higher frequencies of *Streptomyces* inhibitory phenotypes of the two plant pathogens, *Streptomyces scabies* and *Fusarium oxysporum*, were observed in response to soil carbon additions. Additionally, to determine if shifts in *Streptomyces* functional characteristics correlated with microbiome composition, we investigated whether shifts in functional characteristics of soil *Streptomyces* correlated with composition of soil bacterial communities, analyzed using 16S rRNA gene sequencing. Regardless of dose, community composition differed significantly among carbon-amended and non-amended soils from both sites. Carbon type and dose had significant effects on bacterial community composition in both LOM and HOM soils. Relationships among microbial community richness (observed species number), diversity, and soil characteristics varied among soils from different sites. These results suggest that manipulation of soil resource availability has the potential to selectively modify the functional capacities of soil microbiomes, and specifically to enhance pathogen inhibitory populations of high value to agricultural systems.

## Introduction

Soil organic amendments are an increasingly popular practice used to enhance soil health and quality in managed agroecosystems (Gómez-Sagasti et al., 2018). Green manures, composts, and crop residues have been used broadly as means to increase soil organic matter content and carbon availability, and consequently, stimulate microbial biomass and activity in the soil (Larkin, 2015; Francioli et al., 2016). For example, addition of organic amendments to soil has been associated with natural suppression of soilborne plant pathogens (Larkin, 2015; Liu et al., 2015; Jaiswal et al., 2017). Possible mechanisms of suppression include the increase in microbial antagonism, as well as plant growth promotion, activation of systemic induced resistance, and accumulation of plant-derived biocidal compounds (Zhang et al., 1998; Wiggins and Kinkel, 2005b; Bressan et al., 2009). However, the complexity and heterogeneity of organic amendments that are normally used in agriculture limit our capacity to elucidate whether the intensification of pathogen suppression in the soil occur directly from the carbon inputs in the amendment. In this study, we explore the effects of single purified carbon compounds constituents of both plant root exudates and commonly used organic amendments on soil microbiome composition, and specifically their capacities to selectively enrich pathogen antagonistic communities in soil.

*Streptomyces* are Gram positive, filamentous bacteria that are ubiquitous in the soil, and known as prolific producers of a broad range of antibiotics used both as antibacterial and antifungal compounds (Sánchez et al., 2010; Seipke et al., 2012; Viaene et al., 2016). In agricultural settings, *Streptomyces* species appear to be promising biocontrol agents due to their antagonistic activity and ability to suppress diverse plant pathogens (Samac and Kinkel, 2001; Xiao et al., 2002; Otto-Hanson et al., 2013; Law et al., 2017). Nutrient competition is considered a major driver of antagonistic competitive interactions among soil *Streptomyces* (Kinkel et al., 2014). Manipulation of soil carbon availability through addition of organic amendments has been used in attempts to deliberately enrich the densities and antagonistic activity of soil *Streptomyces*. For example, green manures applications and crop rotations have been associated with enhanced pathogen suppressive activity of soil *Streptomyces* (Wiggins and Kinkel, 2005a,b; Vaz Jauri et al., 2018). Similarly, simple carbon substrates have been shown to influence *Streptomyces* metabolic capacities and antibiotic inhibitory interactions in agricultural (Dundore-Arias et al., 2019) and prairie soils (Schlatter et al., 2009). However, the direct selective effects of carbon inputs on *Streptomyces* pathogen antagonism, and the links between shifts in *Streptomyces* community characteristics and bacterial community composition in the soil remain unknown.

Soil organic amendments can alter microbiome composition, structure, and activity (Hartmann et al., 2015; Zhalnina et al., 2015; Francioli et al., 2016). Previous studies have linked shifts in microbiome structure and functional diversity with suppression of plant pathogens in soils treated with organic amendments (Liu et al., 2015; Jaiswal et al., 2017). Organic amendments have therefore been proposed as a means to manage soil indigenous microbial communities and promote suppression of diseases caused by soilborne pathogens in agricultural systems (Gómez Expósito et al., 2017). However, the use of complex organic amendments to promote microbial activity and intensify soil suppressiveness against plant pathogens has often been inconsistent (Wiggins and Kinkel, 2005a,b; Bonanomi et al., 2010; Liu et al., 2015; Jaiswal et al., 2017). Deciphering how specific carbon inputs influence the selection of key microbial groups and functional traits will be critical to understand the mechanisms that lead to the onset of the pathogen suppressive condition.

In this study, we investigated the effects of carbon inputs on microbiome composition and function in agricultural soils. Specifically, we evaluated the changes in non-plant pathogenic soil *Streptomyces* (hereafter referred to as *Streptomyces*) densities and pathogen suppressive capacities, as well as soil bacterial community composition, in response to repeated additions of carbon compounds to soil. We hypothesized that carbon amendments would impose selection that would influence *Streptomyces* densities and pathogen suppressive capacities. Additionally, we postulated that shifts in *Streptomyces* community characteristics would correlate with changes in the relative abundance of other bacterial taxa. Broadening our understanding of the effect that manipulation of soil resource availability via carbon amendments play in mediating the composition and functional capacities of soil microbiomes will allow us to design tailored strategies to enhance pathogen inhibitory microbial communities in agroecosystems.

## Materials and Methods

### Soil Collection and Processing

Soil was collected in October 2012 from two freshly-tilled field sites in Minnesota: the University of Minnesota (UMN) Outreach, Research and Education Park (Rosemount, MN), and the UMN Becker Sandplain Research Farm (Becker, MN). The soil from the Rosemount site (hereafter referred to as high organic matter, HOM) was a Waukegan silt loam (fine-silty over sandy or skeletal, mixed mesic Typic Hapludoll) obtained from border rows in which the primary vegetation was red clover (*Trifolium pratense*), and contained an average organic matter of 4.8 % and nitrate of 10.3 ppm. The soil from the Becker site (hereafter referred to as low organic matter, LOM) was a Hubbard loamy sand (mixed, frigid Psammentic Hapludalf) with a history of cultivation in potatoes (*Solanum tuberosum*), and contained an average organic matter of 1.5% and nitrate of 15.2 ppm. Organic matter and nitrate content were analyzed at the University of Minnesota Research Analysis Laboratory on subsamples of ~250 g of soil from the bulked samples collected from each site.

### Mesocosm Establishment

Soil mesocosms were established as previously described (Dundore-Arias et al., 2019). Briefly, sterile 1 pint (0.47 L) canning jars were filled with 500 g dry weight of sieved and homogenized soil, and covered with two layers of sterile muslin cloth held on by a screw-cap metal ring to reduce the risk of microbial contaminants, but allowing free gas exchange. Mesocosms were arranged in a randomized complete block design, and incubated in a dark, dehumidified space at ~25°C. Soils were incubated for ~1 month before the addition of carbon substrates to reduce labile carbon prior to imposing the treatments.

Soil in each jar was amended with a low or high dose sterile solution of glucose (Sigma-Aldrich Co, St. Louis, MO, USA); fructose (Fisher Scientific Co., Pittsburgh, PA, USA); malic acid (Acros Organics, Morris Plains, NJ, USA); a mixture amendment consisting of a combination of glucose, fructose, and malic acid; or with only sterile water (non-amended control). There were nine treatments applied to soils from each site, and four replicated mesocosms for each treatment, resulting in a total of 72 mesocosms including the non-amended controls.

The carbon compounds used here were selected because they represented substances reported to be dominant constituents of plant litter and root exudates (Schutter and Dick, 2001; Dakora and Phillips, 2002; Orwin et al., 2006; Bradford et al., 2008). The low dose (equivalent to 250 g C/m^2^/year) amendment was chosen to approximate the annual carbon input to a highly productive prairie soil, and the high dose (equivalent to 750 g C/m^2^/year) to exceed this amount (Hernández and Hobbie, 2010). For the complex carbon amendment, an adjusted amount of each of the three substrates was added so that each comprised an equal fraction of the total added carbon in that treatment. Amendments were made once every 14 days for the first 3 months, and once per month thereafter, with equal amounts of carbon added to each soil mesocosm each time. At every amendment time, soil moisture was adjusted (3.0 bars of tension) to maintain consistency following previously described methods (Dundore-Arias et al., 2019). Non-amended control mesocosms received an equivalent volume of sterile water, which in general ranged from 23 to 37 mL at each time point, based on soil moisture. For all the experiments described here, soil samples were taken at the end of the 9 months incubation period (1 month after the last carbon amendment).

### Streptomyces Density Assessment

Densities of *Streptomyces* spp. were estimated using a modified Herr's method (Herr, 1959 as described in Wiggins and Kinkel, 2005a). Specifically, 1 g of soil was taken from each mesocosm and dried overnight in a fume hood beneath four layers of sterile cheesecloth at ~25°C. Soil was then added to 20 mL of sterile water, and shaken for 60 min at 175 rpm and 4°C. The soil suspensions were serially diluted, and 0.1 mL of the target dilution was spread onto plates containing 15 mL of 1% water agar (WA). Inoculated plates were immediately overlaid with 5 mL of the *Streptomyces*-selective medium, starch casein agar (SCA; Becker and Kinkel, 1999), and incubated for 3 days at 28°C. This double layer agar method (WA/SCA) is selective predominantly for *Streptomyces*, whose densities (CFU/g of soil) were estimated by counting all visible colonies on each plate based on colony morphology.

### *In vitro* Inhibition Assay

The frequency and intensity of inhibitory *Streptomyces* phenotypes against the plant pathogens *Fusarium oxysporum* (strain 11390 provided by John Leslie at Kansas State University, hereafter referred to as *Fo*,) and *Streptomyces scabies* (strain S87 isolated by D. Liu at the University of Minnesota, hereafter referred to as *Ss*,) were assessed in dilution plates used to estimate densities of *Streptomyces* (described above). Assays were conducted as previously described (Felice et al., 2014). Briefly, dilution plates were overlaid with either a *Fo* or *Ss* culture. Specifically, *Fo* culture plates were cut into 1 cm squares and blended until smooth with 100 mL sterile deionized water in a sterile blender (Waring Laboratory Science, Torrington, CT, USA). The resulting mixture (10 mL) was combined with 250 mL Potato Dextrose Water Agar (PDWA; 1.2 g Bacto Potato Dextrose Broth and 10.0 g Bacto Agar per liter of deionized water) and 10 mL of the final *Fo*-PDWA mixture were overlaid onto each dilution soil plate and incubated at 25°C for 3 days. In the case of *Ss* overlays, soil dilution plates were overlaid with 10 mL of SCA, which was allowed to solidify. A 150 μL aliquot of a suspension of *Ss* (~10^8^ CFU/mL) in 20% glycerol was then added to each plate and spread over the SCA surface. The overlaid plates were incubated for 3 days at 28°C. After the incubation period, densities and frequencies of pathogen-inhibitory interactions were assessed by counting the number of colonies that inhibited completely the growth of the overlaid pathogen around the colony. Colonies that only partially inhibited pathogen growth were not included. Intensities of pathogen-inhibitory interactions were assessed by measuring individual inhibition zones. Specifically, two perpendicular measurements were taken from the colony boundary to the edge of the cleared inhibition zone, and averaged to provide a single measurement for every colony.

### Soil Chemistry

Soil samples were collected from each mesocosm, and were air-dried for pH analysis or oven-dried (60°C) for analysis of total carbon (TC), total nitrogen (TN). Soil pH was measured following standard methods (Watson and Brown, 1988), and using a water pH meter (Mettler Toledo, Columbus, OH, USA) on a 1:1 suspension. Soil TC and TN were determined at the University of Nebraska Ecosystem Analysis Laboratory (Lincoln, NE) using dry combustion analysis by a Costech Analytical ECS 4010 (Costech Analytical Technologies Inc., Valencia, CA, USA).

### Amplicon Sequencing

A total of 27 soil samples (9 treatments × 3 mesocosms per treatment) from each site were collected for amplicon sequencing analysis and stored frozen (−80°C) until further analysis. DNA was extracted from ~0.25 g of each soil using MoBio PowerSoil DNA Extraction kits (MO BIO Laboratories Inc., Carlsbad, CA, USA). Soil was added to the PowerBead tubes and sonicated for 10 min, and the standard manufacturer's protocols for bulk DNA extractions were followed. Extracted DNA was submitted to the University of Minnesota Genomics Center (http://genomics.umn.edu/) for library preparation and Illumina MiSeq sequencing of the V5–V6 hypervariable regions of the 16s ribosomal RNA gene.

PCR amplification was conducted for the marker gene region (16S V5–V6), in triplicate, using KAPA HiFidelity Hot Start Polymerase (Gohl et al., 2016a,b). The first PCR-amplification were achieved using the V5F and V6R Nextera primer pair (TCGTCGGCAGCGTCAGATGTGTATAAGAGACAGRGGATTAGATACCC and GTCTCGTGGGCTCGGAGATGTGTATAAGAGACAGCGACRRCCATGCANCACCT, Staley and Sadowsky, 2016). PCR conditions were as follows. Samples were heated for 5 min at 95°C, followed by 25 cycles of 98°C for 20 s, 55°C for 15 s, 72°C for 1 min, and finally samples were held at 4°C. A second 10-cycle PCR amplification step, using the same cycle conditions, was used to add forward and reverse indexing primers to 5 μl of 1:100 PCR product from the first round of PCR. For all amplicon sequences, pooled, size-selected samples were denatured with NaOH, diluted to 8 pM in Illumina HT1 buffer, spiked with 15% PhiX DNA, and heat denatured at 96°C for 2 min immediately prior to loading. A MiSeq 600 cycle v3 kit was used to sequence samples on an Illumina MiSeq instrument (Illumina, San Diego, CA, USA).

### Amplicon Sequencing Data Processing

Raw sequence data were processed as follows. Quality control and trimming/filtering were performed based on per-base and per-read quality scores. PandaSeq was used to connect overlapping paired-end reads, while non-overlapping paired-end reads were treated as single-end reads (Masella et al., 2012). Non-overlapping reads had the 3′ ends trimmed based on quality. ChimeraSlayer's usearch61 method was used to detect and remove chimeras (Haas et al., 2011). OTU picking was conducted using QIIME's pick_open_reference_otus.py script with the usearch61 method (Caporaso et al., 2010). The resulting OTU table was filtered to remove any sequences that could not be identified as bacterial, and mitochondrial and chloroplast sequences were removed. Taxonomic assignments were done at 97% similarity using the Greengenes (DeSantis et al., 2006; McDonald et al., 2012; http://greengenes.secondgenome.com) release 13_8 database. Post-pipeline data processing and analysis were done using QIIME 1.8 (Caporaso et al., 2010). Alpha diversity was calculated as bacterial diversity (Shannon H′ Index) and OTU richness (number of distinct OTUs) on a per sample basis using QIIME. Bacterial libraries were rarefied to the uniform depth of 5,000 sequences per sample using multiple rarefaction prior to analyses of alpha diversity. Three samples (two from high malic acid-high dose, and one from mixture-high dose) were excluded from all analyses due to low sequence depths (<1,000 sequences/sample).

### Data Analyses

Significant differences in soil chemical and microbial metrics between LOM and HOM soils were observed before and after mesocosm establishment, and therefore, data from each soil type were analyzed and are presented here separately. All data analyses were conducted using R statistical software R version 3.2.0 (R Core Team, 2014). Total colony counts of *Streptomyces*, and counts of inhibitory *Streptomyces* were used to calculate densities of total and inhibitory *Streptomyces*. In order to avoid taking the log of 0, in the cases of no inhibitory activity, one inhibitor colony was added to each plate count across all treatments. Density calculations were determined as Log CFU/g of soil. Mean inhibition zone (hereafter referred to as mean killing zone, MKZ), proportions of inhibitors and percent changes from control were calculated using the following formulas:

$$\begin{array}{l} \text{Mean inhibition zone for MKZ}\\ =\text{Sample average }\left[colony\;inhibition\;zone\;\left(mm\right)/plate\right]\\ \text{Proportions of inhibitors}\\ =\text{Average}\frac{Count\;of\;pathogen\;inhibitory\;Streptomyces\;phenotypes}{Count\;of\;total\;Streptomyces}\\ \text{Percent change}\\ =\frac{Mean\;control\;value-Mean\;treatment\;value}{Mean\;control\;value} \end{array}$$

Proportions were arcsine-square root transformed prior to analysis. Percent changes from control indicate the percentage of increase (positive values) or decrease (negative values) in soil chemical and *Streptomyces* community characteristics in response to carbon amendments. Analysis of variance (ANOVA) and least significant difference test (LSD), both with *p* < 0.05 as the significance level, were used to analyze differences in soil chemical characteristics, *Streptomyces* density and pathogen-inhibitory capacities, and bacterial community diversity among treatments. Additionally, Pearson's correlation was performed using the R's corrplot package (Wei and Simko, 2016) to characterize the relationships between soil chemistry and *Streptomyces* populations.

Microbiome Beta diversity was assessed on Hellinger transformed OTU-based Bray-Curtis distances (BC-OTU) using non-metric multidimensional scaling (NMDS) in the metamds function of the Vegan package for R, version 3.2.0 (Oksanen et al., 2015). Significant differences in community composition among carbon substrates and doses were assessed using permutational analysis of variance on distance matrices (PERMANOVA; Anderson, 2001) with the Adonis function in vegan (Oksanen et al., 2017) for each soil type. Relationships between bacterial community structure and soil chemistry, and metrics of *Streptomyces* populations were tested using Vegan's envfit function (999 permutations) (Oksanen et al., 2017) and *p*-values were adjusted to account for multiple tests using a Bonferroni correction (*p*_adj_). Relationships between microbial communities (richness, diversity, relative abundances of different phyla) and soil chemical characteristics and metrics of *Streptomyces* populations were explored using Spearman's Rank correlations in R's cor package using the “complete pairwise observation” option.

## Results

### Carbon Amendments Induced Changes in Soil Chemical Properties

**Figure 1:**
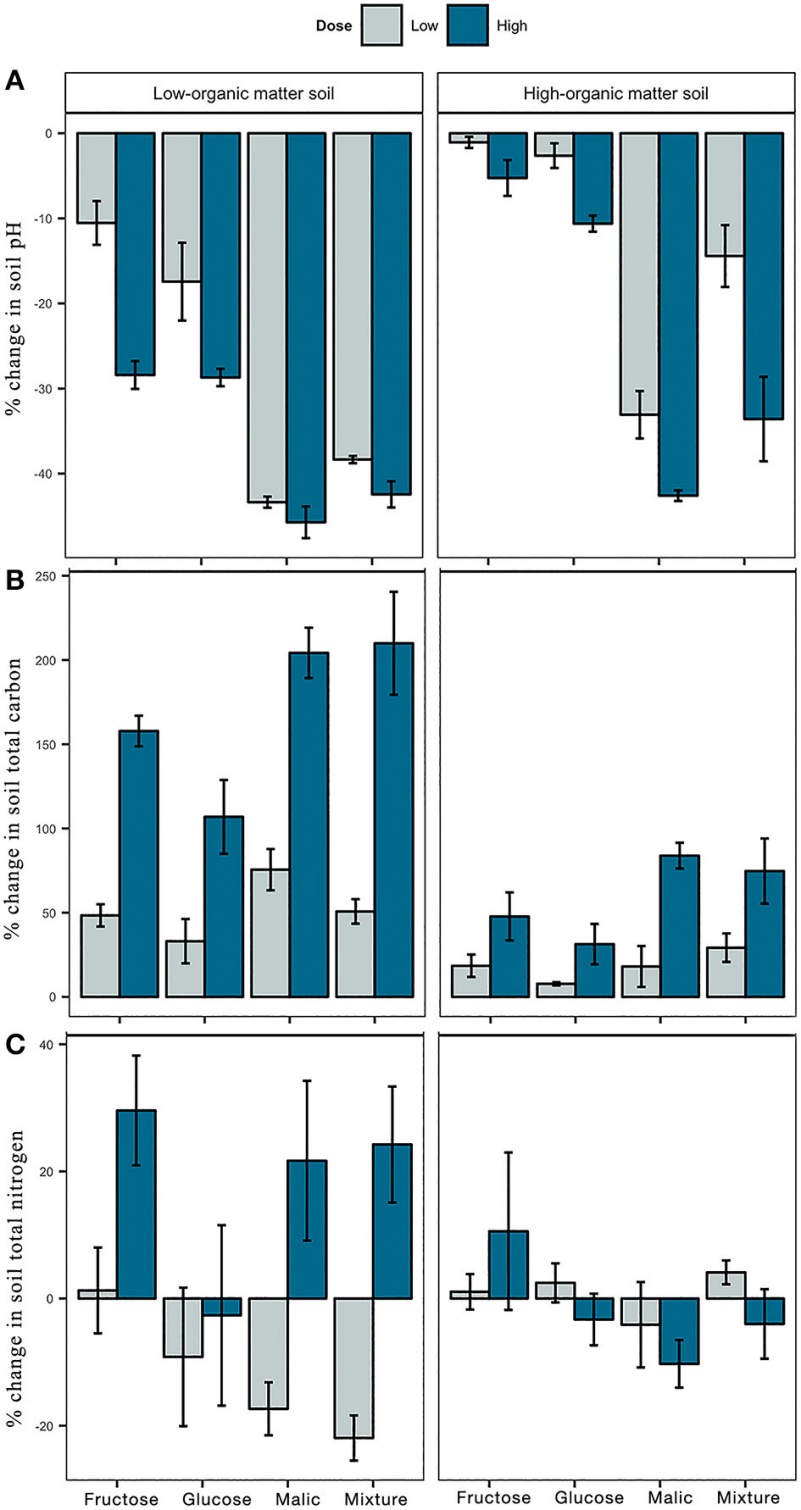
Effect of carbon amendments on chemical characteristics of low- and high-organic matter soils, including pH **(A)**, and total carbon **(B)**, and total nitrogen **(C)** content. Each bar represents the mean percent change from non-amended controls. Positive and negative values indicate the percentage of increase or decrease from control, respectively. Error bars represent ± 1 standard error from the mean.

Soil pH in mesocosms declined substantially during the 9-month incubation period in amended and non-amended soils (Figure 1A). Regardless of the initial organic matter content in LOM vs. HOM soils, addition of malic acid or the carbon mixture and high dose treatments resulted in greater pH reductions than amendments with fructose or glucose, or a low dose of any substrate (Figure 1A, Tables S1, S2).

Contrary, addition of carbon significantly increased soil TC relative to the control in both LOM and HOM soils (Figure 1B). In both LOM and HOM soils, high dose carbon inputs increased TC more than low dose inputs (Table S1). Although there was a consistent increase in TC between low and high dose treatments in both soils, the specific increase relative to control was greater in LOM (low = 50%, high = 170%) than HOM (low = 18%, high = 60%) soils (Table S2). Finally, soil TN was also influenced by carbon additions (Figure 1C). In LOM but not HOM soil, the majority of high dose carbon inputs significantly increased soil TN relative to control and low dose treatments (Table S2).

### Carbon Amendments Influenced Density and Frequency of Pathogen Inhibition by Streptomyces Populations

**Figure 2:**
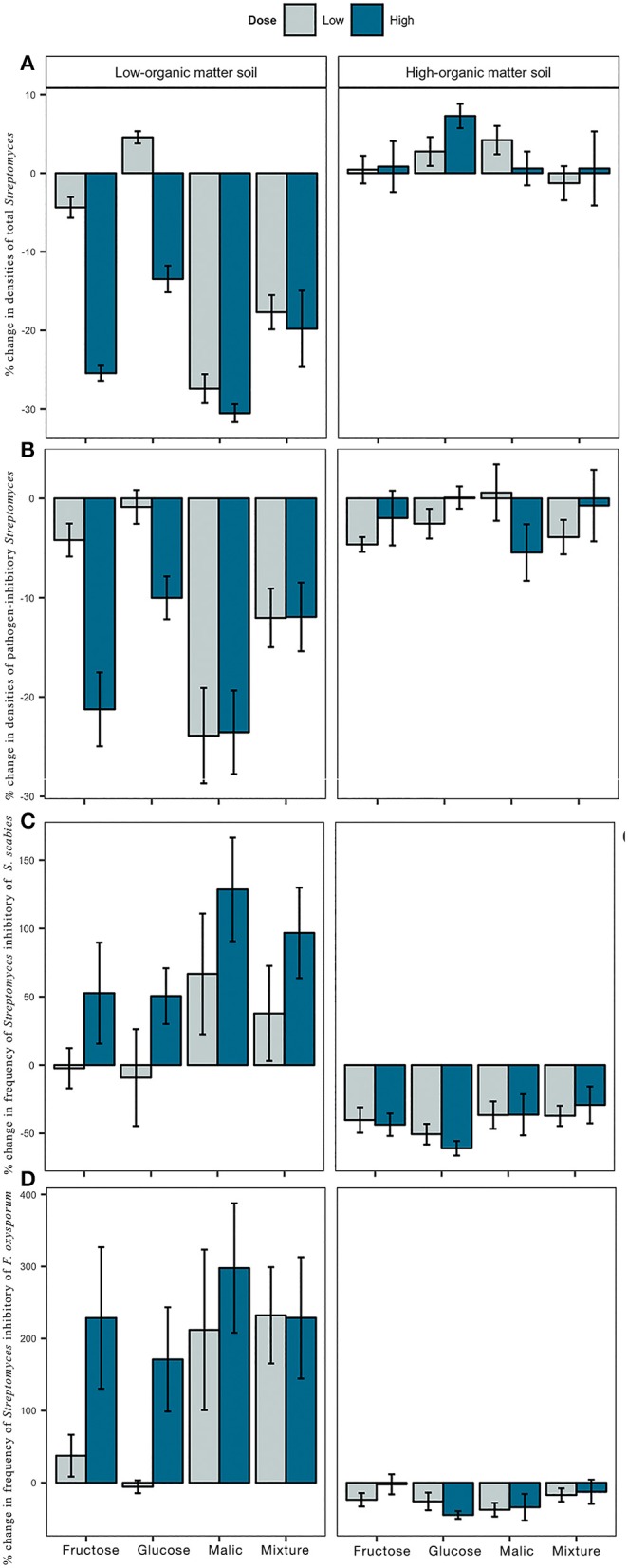
Effect of carbon amendments on *Streptomyces* community characteristics of low- and high-organic matter soils, including densities of total **(A)** and pathogen-inhibitory **(B)** *Streptomyces*, and frequency of *Streptomyces* inhibitory of *Streptomyces scabies-Ss* **(C)** and *Fusarium oxysporum-Fo* **(D)**. Each bar represents the mean percent change from non-amended controls. Positive and negative values indicate the percentage of increase or decrease from control, respectively. Error bars represent ± 1 standard error from the mean.

Impacts of carbon additions to soil on total *Streptomyces* densities varied between LOM and HOM soils (Figure 2A). In LOM soils, significant differences in *Streptomyces* densities were observed among carbon treatments (type, F = 40.77, *p* < 0.05; dose, *F* = 53.13, *p* < 0.05; Table S1). Reductions of 13–30% of *Streptomyces* densities from the control were observed in LOM soils in response to all high dose carbon amendments, and low dose malic acid or carbon mixtures (Figure 2A, Table S2). In contrast, carbon additions in HOM soils resulted in small but non-statistically significant increases in *Streptomyces* densities (1–7%) compared to the control Table S1).

Densities of inhibitory *Streptomyces* were also affected by the carbon amendments tested (Figure 2B). In both LOM and HOM soils, carbon amendments significantly reduced the densities of inhibitory *Streptomyces* relative to control. There were significant differences in inhibitory densities among carbon treatments in LOM (type, *F* = 10.74, *p* < 0.05; dose, *F* = 8.19, *p* < 0.05; Table S1), but not in HOM soils. The strongest negative effect of carbon on densities of inhibitory *Streptomyces* in comparison to the non-amended control was observed in LOM soils, and particularly, in response to additions of malic acid and all high dose carbon amendments (Table S2).

Carbon inputs had the opposite effect on frequencies of pathogen-inhibitory *Streptomyces* in LOM vs. HOM soils (Figures 2C,D). Addition of carbon inputs to soil increased the frequency of inhibitory *Streptomyces* of bacterial (*Ss*, Figure 2C) and fungal (*Fo*, Figure 2D) pathogens relative to the control in LOM soils, but decreased the frequency of pathogen-inhibitory *Streptomyces* in HOM soils. Dose, but not type of carbon input, had significant effects on frequencies of inhibitory *Streptomyces* of *Ss* and *Fo* in LOM soils (*Ss, F* = 5.87, *p* < 0.05; *Fo, F* = 4.36, *p* < 0.05; Table S1), with greater frequencies observed in high dose treatments. Finally, inhibitory *Streptomyces* in carbon-amended LOM soil (but not HOM) soils, tended to produce larger MKZ against both pathogens than their counterparts from non-amended soils (Figure S1).

### Relationships of Soil Chemical Characteristics to Streptomyces Densities and Pathogen Suppressive Capacities

**Figure 3:**
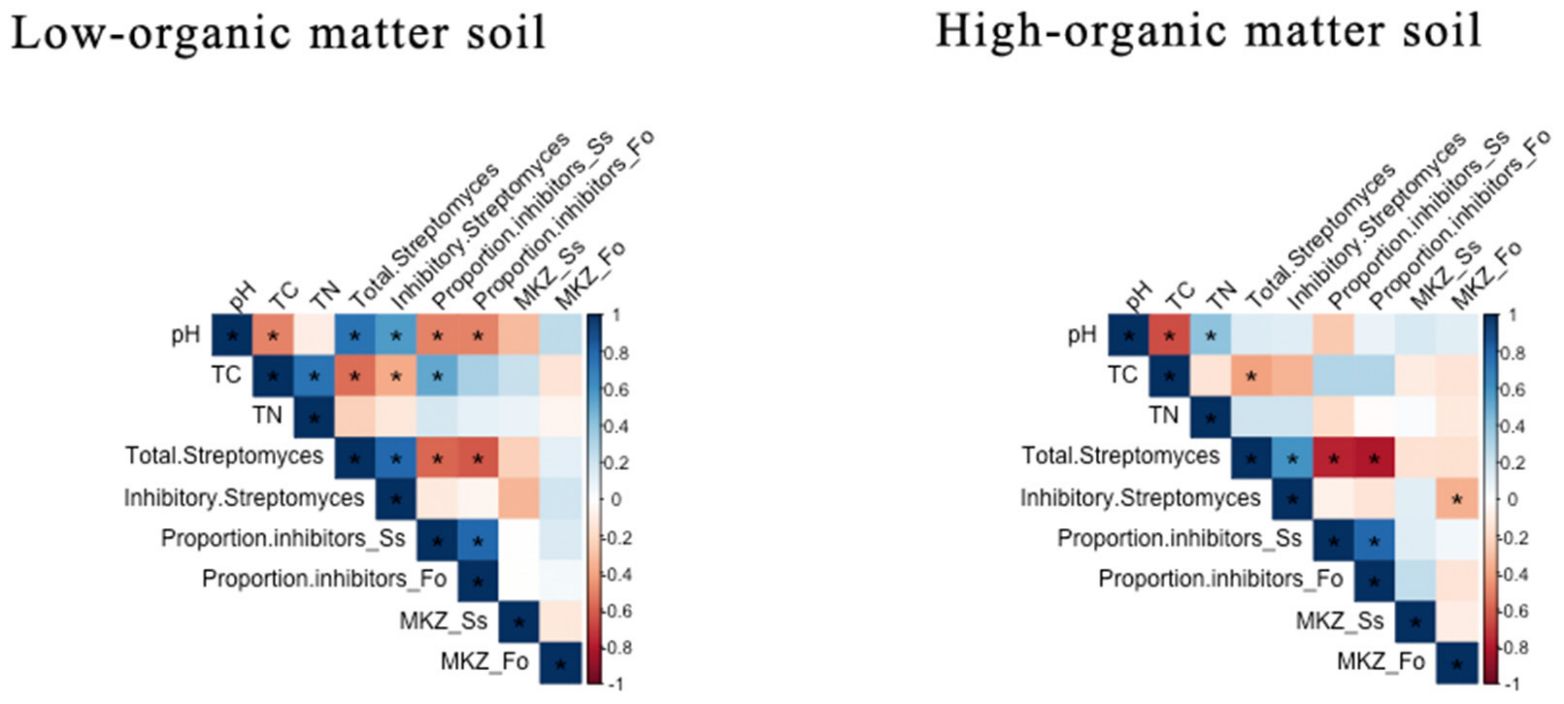
Pearson's correlation analysis of soil chemical and *Streptomyces* community characteristics in low- and high-organic matter soils. The direction and strength of the correlations are indicated by color (positive = blue or negative = red). Significant correlation coefficients (*p* < 0.05) are identified with an asterisk. Pearson's correlation coefficient values are reported in Table S3.

In both LOM and HOM soils, pH and total densities of *Streptomyces* were significantly negatively correlated with TC (Figure 3). In both LOM and HOM soils, pH was positively and negatively correlated with densities and proportion of pathogen-inhibitory *Streptomyces*, respectively. However, these relationships were significant only in LOM soils. Overall, these results indicate that carbon amendments have considerable impacts on soil *Streptomyces* populations, and can select for increased frequencies of inhibitory populations under conditions of increasing TC and decreased pH.

### Carbon Amendments Had Significant Effects on Bacterial Community Composition and Diversity

**Figure 4:**
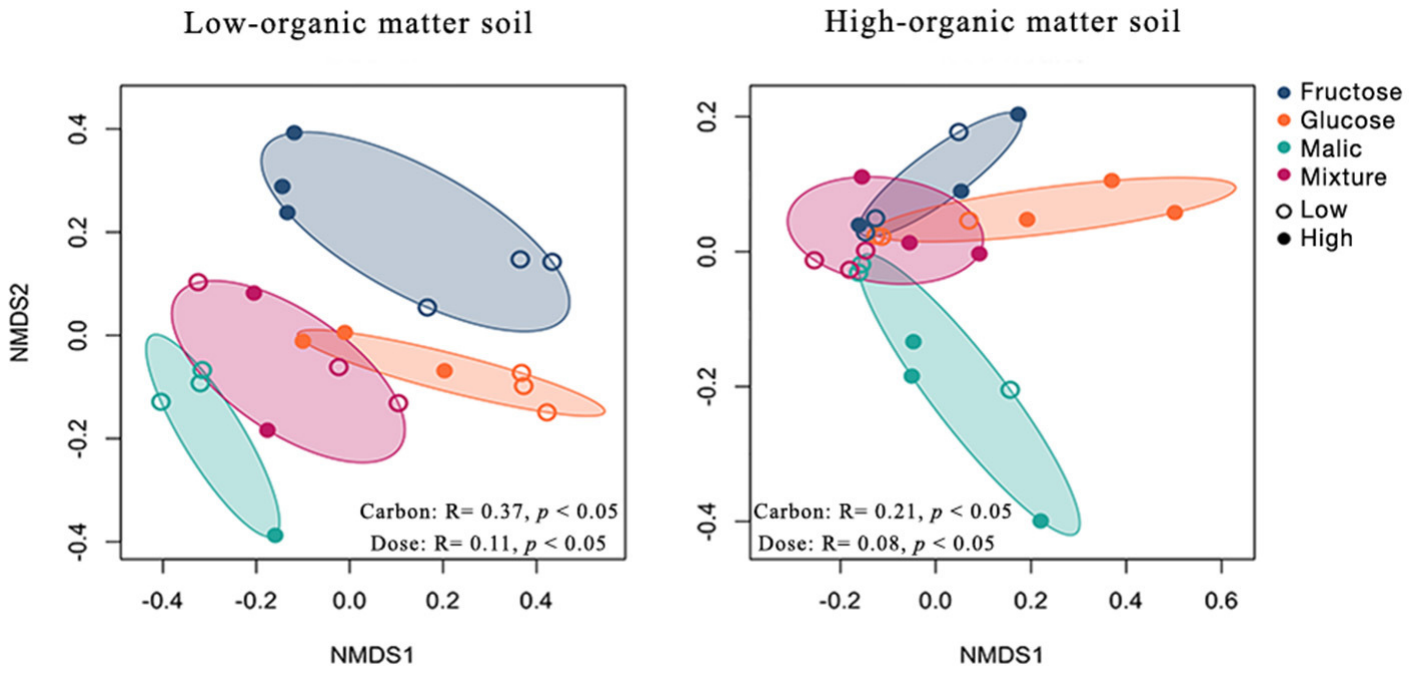
Effect of carbon amendments on bacterial communities in low- and high-organic matter soils. Non-metric multidimensional scaling (NMDS) ordination of Hellinger transformed Bray-Curtis dissimilarities of operational taxonomic unit (OTU) counts. Points represent individual samples from mesocosms amended with low (open circles) or high (solid circle) doses of fructose (blue), glucose (orange), malic acid (green), and a mixture of these substrates (pink). Significant differences in community composition among carbon amendment type and dose were assessed using permutational analysis of variance on distance matrices (PERMANOVA). *Adonis R*^2^ and *p*-values are indicated in the lower part of each plot. NMDS stress values were <0.20 in all cases.

Overall, community composition varied significantly between carbon-amended and non-amended LOM (PERMANOVA, *R*^2^ = 0.28, *p* < 0.05) and HOM (PERMANOVA, *R*^2^ = 0.18, *p* < 0.05) soils (Figure S2). In both LOM and HOM soils, mesocosms amended with different carbon substrate types harbored significantly different bacterial communities (PERMANOVA; LOM, *R*^2^ = 0.37, *p* < 0.05; HOM, *R*^2^ = 0.21, *p* < 0.05; Figure 4). Further, carbon amendment dose significantly correlated with differences in mesocosm bacterial communities in both soils (PERMANOVA; LOM, *R*^2^ = 0.08, *p* < 0.05; HOM, *R*^2^ = 0.11, *p* < 0.05; Figure 4).

In LOM soils, community composition was significantly related to soil pH, as well as densities of total *Streptomyces*, and densities and frequency of pathogen-inhibitory *Streptomyces* (Figure S3). In HOM soils, variation in bacterial community composition was significantly related to soil pH, but not to *Streptomyces* community characteristics (Figure S3).

**Figure 5:**
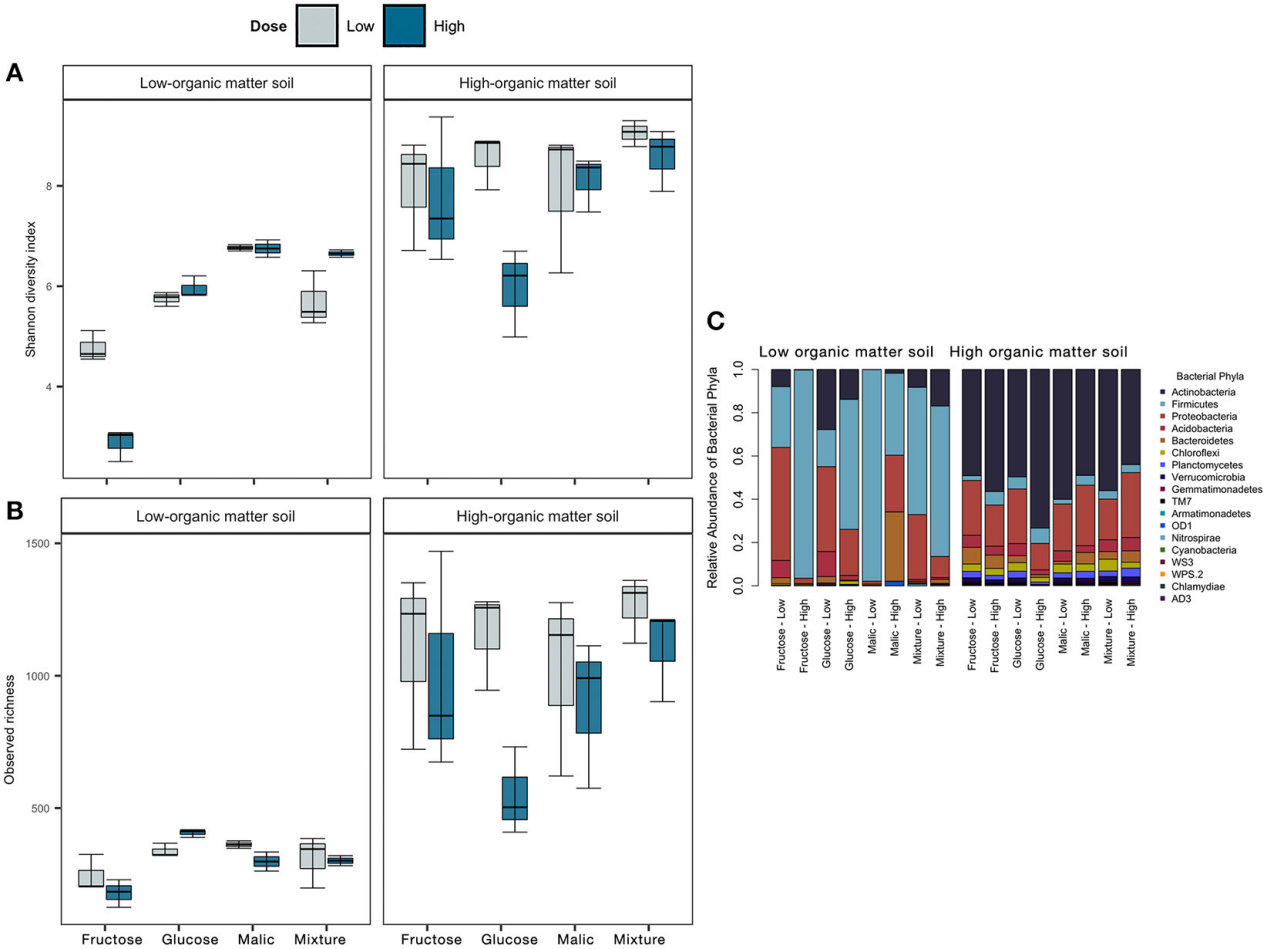
Effect of carbon amendments on bacterial community alpha diversity (Shannon H' index, **A**) and observed OTU richness **(B)**, and relative abundances of major bacterial phyla **(C)** in low- and high-organic matter soils. In **(A,B)**, lines inside the boxes represent the median, while the whiskers represent ± 1 standard error.

Carbon amendment type and dose had significant effects on bacterial community alpha (within-sample) diversity and richness in both LOM and HOM soils (Figures 5A,B). In LOM, but not HOM soils, significant differences were observed for bacterial diversity and richness estimators among different carbon substrate types (Shannon index, *F* = 92.43, *p* < 0.05; observed richness, *F* = 8.76, *p* < 0.05, Table S1). In contrast, dose had a significant effect only on bacterial richness in HOM soils (*F* = 5.02, *p* < 0.05; Table S1).

We compared the relative abundance of dominant bacterial phyla from mesocosms amended with different carbon substrate types (Figure 5C). Relative abundance of the dominant bacterial phyla varied markedly between LOM and HOM soils, and in both cases, we observed a reduction in the number of phyla represented between control and carbon-amended mesocosms (Figure S4). Relative to control, there was an increase in relative abundances of Firmicutes and Actinobacteria in LOM and HOM soils, respectively, and these phyla were also the most predominant post-carbon amendment in each soil. Within those phyla, greater relative abundances of members of the order Bacillales and Actinomycetales were observed in LOM and HOM soils, respectively (Figures S5A,B). Moreover, the relative abundance of Acidobacteria, Chloroflexi, Planctomycetes, Verrucomicrobia decreased, relative to the control treatments, in both LOM and HOM soils.

In LOM soils, bacterial community composition varied consistently among soils amended with a given carbon substrate regardless of the dose. With the exception of malic acid, Firmicutes were more abundant in high than low dose treatments, while relative abundance of Proteobacteria and Actinobacteria were greater in soils amended with low dose treatments. Fewer differences among carbon-amended soils were observed in HOM soils, although, it was observed that Actinobacteria and Firmicutes had greater relative abundances in high vs. low dose fructose and glucose treatments. Together, these results show that carbon amendments significantly alter soil bacterial community composition, and can selectively enrich bacterial taxa in ways that depend both on the carbon substrate type and dose.

### Relationships of Soil Chemistry and Streptomyces Populations and Abundance of Bacterial Phyla in Carbon-Amended Soils

**Figure 6:**
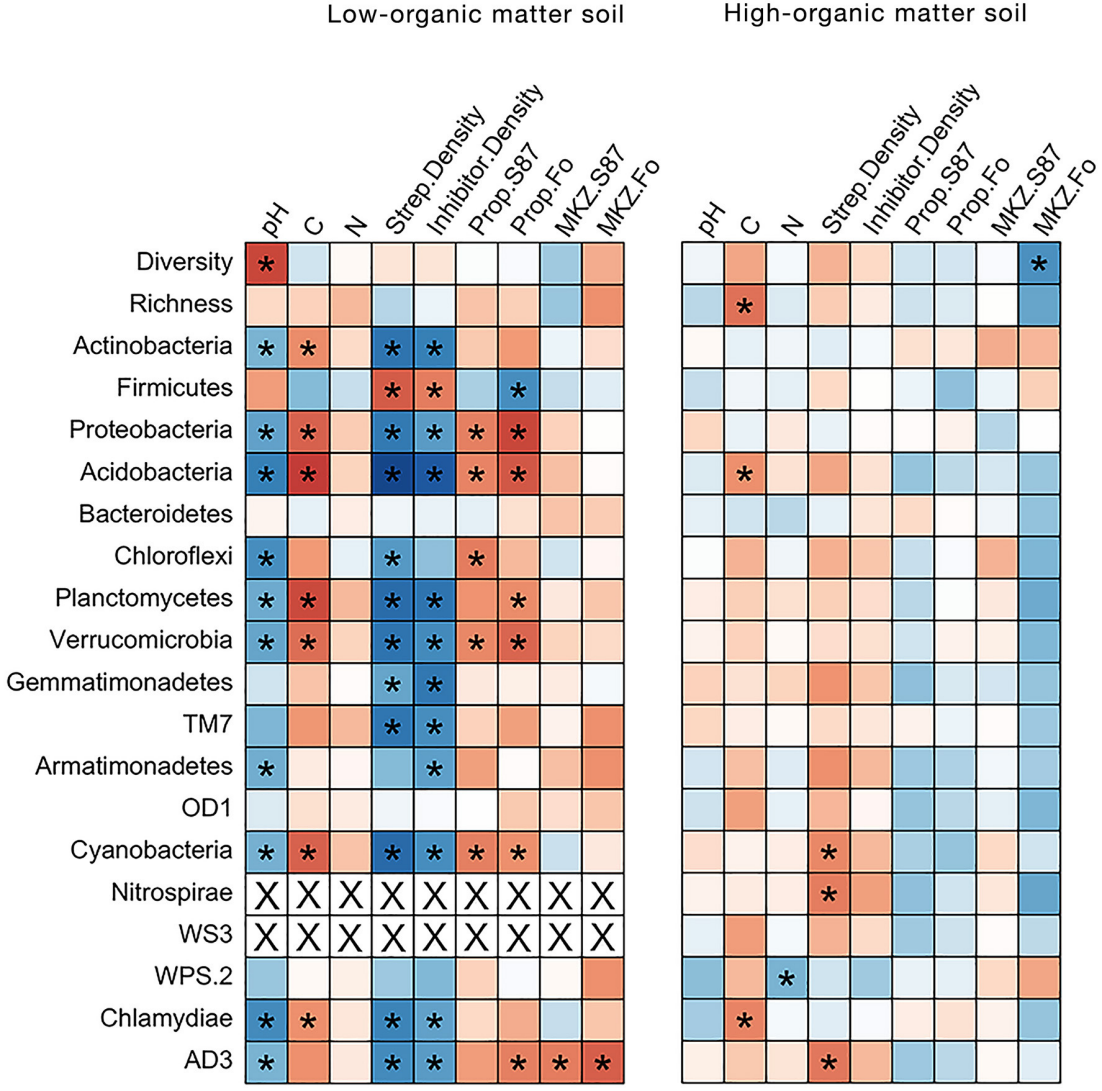
Heatmap of Spearman's rank correlation coefficients between relative abundance of bacterial phyla and soil chemical and *Streptomyces* community characteristics in low- and high-organic matter soils. The direction and strength of the correlations are indicated by color (positive = blue or negative = red). Significant correlation coefficients (*p* < 0.05) are identified with an asterisk. Spearman's rho coefficient values are reported in Table S4.

In LOM soils, there was a strong negative correlation between soil pH and bacterial community diversity (Figure 6), as well as between soil pH and TC and relative abundance of many major bacterial phyla. *Streptomyces* densities were significantly positive correlated with relative abundances of Actinobacteria, Proteobacteria and Acidobacteria, while abundances of the latter two phyla were significantly negatively correlated with the proportions of inhibitory *Streptomyces* (Figure 6). Positive relationships were observed between the relative abundance of multiple phyla (Planctomycetes, Verrucomicrobia, Cyanobacteria, Chlamydiae, and candidate divisions TM7 and AD3) and *Streptomyces* densities, while relative abundances of Firmicutes were negatively correlated with densities of *Streptomyces* (but positively correlated with the proportions of inhibitory *Streptomyces*). By contrast, in HOM soils, no significant correlations between bacterial community and soil and *Streptomyces* community parameters were observed. Overall, these data demonstrate that soil carbon amendments influence soil bacterial communities, and for certain phyla, these effects are also linked to soil chemistry and *Streptomyces* population characteristics.

## Discussion

Carbon inputs to soil mesocosms induced significant changes in soil chemistry, reducing pH and increasing TC relative to non-amended soils. The magnitude of effects of carbon amendments on pH and TC varied between LOM and HOM soils, likely as a function of differences in intrinsic soil properties (Helling et al., 1964; Lauber et al., 2008; Liao et al., 2016). Although both soils received the same low and high doses of carbon substrates, greater percentage increases in TC were observed in LOM than HOM soils after the 9 months incubation. The overall greater TC accumulation in LOM soils may be explained by a phenomenon known as negative priming, characterized by the suppression of existing soil organic matter (SOM) decomposition under low carbon conditions, as soil microbes may preferentially use the labile carbon sources provided in the amendments over the existing more recalcitrant carbon sources (Liu et al., 2017). It is likely that the incubation of soil mesocosms for ~1 month, prior to carbon additions intensified the magnitude of carbon limitation and negative priming in LOM compared to HOM soil. Thus, although the provided labile carbon sources were preferentially used in both soils, the SOM decomposition by the indigenous microbial communities was restrained in LOM soils, resulting in greater TC accumulations.

Similarly, changes in soil chemistry were correlated with responses of bacterial communities to carbon amendments in both LOM and HOM soils. The greater reduction in pH and increase in TC in LOM relative to HOM soils were associated with greater decreases in total *Streptomyces* densities and relative abundances of various bacterial phyla. It is possible that the greater pH reduction, relative to the control in LOM soils (Table S2) produced a less suitable environment for a broad range of microbial groups, including *Streptomyces*, that prefer a neutral to alkaline environmental pH (Hagedorn, 1976; Kontro et al., 2005; Fierer and Jackson, 2006). While carbon amendments decreased pH in both LOM and HOM soils, the greater organic matter content and correspondingly greater buffering capacity, as well as differences in soil texture, in the latter may have alleviated the selecting effect of lowering pH on the bacterial communities in these soils (Naramabuye and Haynes, 2006).

In both soils, there was a strong negative relationship between total densities and proportions of pathogen-inhibitory *Streptomyces*. Nutrient competition is hypothesized to be one of the main drivers of antagonistic interactions among metabolically similar microbial species (Case and Gilpin, 1974). We have little knowledge of the conditions that maximize nutrient competition in soil communities. In earlier work we characterized *Streptomyces* isolates from carbon-amended soils, and demonstrated that shifts in microbial nutrient use and growth capacities coincided with positive selection for *Streptomyces* inhibitory phenotypes in carbon-amended soils (Dundore-Arias et al., 2019). Specifically, carbon inputs selectively enriched *Streptomyces* populations that combine both generalist lifestyle and antagonistic capacities against the strongest resource competitors. In related work, Essarioui et al. (2016, 2017) demonstrated that antagonistic phenotypes were greater among populations of *Streptomyces* and *Fusarium* with highly similar resource use, suggesting significant role for resource competition in selection for antagonism. Here, the simultaneous reduction in *Streptomyces* densities and increase in frequencies of inhibitory *Streptomyces* of both *Ss* and *Fo* in carbon-amended LOM soils suggest the potential for carbon amendments, and the related changes in soil properties and carbon availability, to increase growth and or survival of antagonistic *Streptomyces* populations over non-antagonists. By providing soil microbes with an easily accessible energy supply, carbon amendments may have intensified nutrient competition, increasing biomass and activity of fast-growing *r*-strategists (Ho et al., 2017). Augmentation of nutrient competition may have resulted in an overall decrease in *Streptomyces* densities, and the concurrent selection of the most competitive populations by enabling the biosynthesis of secondary metabolites and signaling molecules to inhibit potential competitors (Hagedorn, 1976; Ratzke and Gore, 2018). Thus, the higher frequency of pathogen-inhibitory phenotypes in soils with increased TC levels in LOM soils suggests that carbon amendments may increase the benefit of maintaining inhibitory traits in environments with greater nutrient competition (Schlatter and Kinkel, 2015). Furthermore, the lowest densities but the highest frequencies of inhibitory *Streptomyces* populations were observed in LOM soils with the lowest pH. These results confirm observations from a previous study that selection for inhibitory phenotypes may be enhanced under biotically or abiotically stressful conditions that are less optimal for growth of *Streptomyces* populations (Otto-Hanson and Kinkel, 2019). Collectively, the enrichment of pathogen-inhibitory populations of *Streptomyces* may reflect shifts in metabolic capacities and diverse species interactions in response to biotic and abiotic changes imposed by the recurrent addition of soil carbon amendments. Future work should focus elucidating how the inverse relationship between densities and proportions of inhibitory *Streptomyces* influence disease suppression under field settings.

Changes in soil chemical properties in response to repeated additions of carbon inputs were also related to changes in the composition and diversity of bacterial communities in comparison to non-amended soils (Figures S2, S4). In line with other studies (Rousk et al., 2009; Zhalnina et al., 2015; Francioli et al., 2016; Wu et al., 2017; Zhang et al., 2017), we found that soil pH and TC were strongly correlated with bacterial community composition in both LOM and HOM soils. Firmicutes, Actinobacteria, and Proteobacteria were the most abundant phyla across all treatments in both soils. These phyla, generally identified as copiotrophic bacteria, have been shown to have high relative abundance under conditions of elevated carbon availability such as the rhizosphere and carbon-amended and fertilized soils (Fierer et al., 2007; Francioli et al., 2016). It is likely that the repeated addition of labile carbon inputs, and the subsequent increases in TC promoted the growth of populations of members of these phyla. On the other hand, lower abundances of Acidobacteria, Chloroflexi, Verrucomicrobia, and Planctomycetes were observed in carbon-amended soils. These phyla are generally classified as oligotrophic due to their ability to grow in nutritionally deficient conditions (Lauber et al., 2008; Ho et al., 2017), and may be less competitive in carbon-rich conditions. Despite the significant increases in TC observed in LOM soils, simultaneous reductions in soil pH may have prevented a broad range of organisms from accessing labile carbon sources or surviving at suboptimal soil pH (Ramirez et al., 2010; Zhalnina et al., 2015). Taken together, our results support previous work indicating that soil carbon amendments have distinct effects on the abundance of oligotrophic vs. copiotrophic organisms (Fierer et al., 2007; Lauber et al., 2008), and that this effect may not be solely based on the direct input of carbon to soil (Francioli et al., 2016; Zhang et al., 2017).

While positively correlated with *Streptomyces* densities, relative abundance of Proteobacteria and Acidobacteria were significantly negative correlated with proportions of pathogen-inhibitory *Streptomyces*. In contrast, the relative abundances of Firmicutes, the most abundant phylum in LOM soils, were negatively correlated with *Streptomyces* densities, but positively correlated with TC and proportions of pathogen-inhibitory *Streptomyces*. Our data may suggest that interactions among populations (e.g., *Streptomyces* and Firmicutes) within the microbiome may mediate microbial responses to carbon inputs. Previous studies have reported the role of plant-derived carbon substrates, including malic acid in attracting plant-beneficial bacteria members of Firmicutes (Rudrappa et al., 2008; Hunter et al., 2014). Collectively, these observations demonstrate that pulses of carbon substrates into the soil can variously enrich or inhibit specific microbial populations of plant-beneficial or -detrimental soil microorganisms. These results also demonstrate the potential for using soil carbon amendments to shape bacterial community composition, harnessing plant-beneficial microbiomes, and enhancing plant pathogen suppression in agricultural soils (Dundore-Arias et al., 2019). However, it is important to remember that under normal conditions, soil microorganisms have access to diverse and dynamic nutrient sources; therefore, future studies should focus on investigating the effect of additional organic compounds (tannins, fatty acids, minerals) and more complex carbon sources (crop residues) on the selection and function of soil microbiomes, and in the presence of the plant host.

Both type and dose of carbon substrate had variable effects on soil properties, as well as *Streptomyces* populations and bacterial community composition. Differences in TC in response to distinct carbon additions may reveal variation in metabolic capacities and preferences for different carbon substrates by microbial populations with distinct life history strategies and functional traits (Ho et al., 2017). For example, the larger TC increases in both LOM and HOM soils treated with a high dose malic acid vs. other substrates may reflect the accumulation of this organic acid due to an overall decreased microbial activity, or lower preferential utilization of this substrate by the indigenous community (Klimek et al., 2016). In contrast, the lower TC levels in high dose glucose treatment in both LOM and HOM soils agree with previous studies reporting higher preference and use efficiency of glucose than other labile substrates by soil bacteria (Thomson et al., 2013), which may have stimulated microbial activity and increased in soil respiration (Liu et al., 2017). The results presented here show that while dose effects the amount of added carbon, substrate identity determines the fate of carbon inputs in the soil, and therefore, both factors should be considered when designing carbon amendment treatments. Moreover, the comparison between LOM and HOM soils demonstrated that responses of the indigenous microbial communities to additions of carbon amendments are not ubiquitous, and that the magnitude and direction of these responses are highly influenced by the intrinsic and modified chemical characteristics of the soil.

In closing, our findings confirm that soil carbon amendments can impose strong selection on indigenous soil microbiomes, and this outcome is the result of a series of biotic and abiotic interactions, and not exclusively due to the direct addition of carbon to soil. The profound selective effect of carbon amendments on soil microbial communities, and mediating microbial evolutionary history and species interactions has the potential to selectively modify the functional capacities of soil microbiomes, and specifically, to accelerate the augmentation of pathogen inhibitory microbial communities of high value to agricultural systems.

## Declarations

The authors declare no conflicts of interest associated with the presented research. Mention of any trade names or commercial products in this article is solely for the purpose of providing specific information and does not imply recommendation or endorsement by the U. S. Department of Agriculture. USDA is an equal opportunity provider and employer. This paper is a joint contribution from the University of Minnesota and the USDA-ARS-Plant Science Research Unit.

## Data Availability Statement

The datasets generated for this study can be found in the NCBI database (BioProject ID: PRJNA576468).

## Author Contributions

JD-A wrote manuscript with significant feedback from co-authors. JD-A and SC analyzed and interpreted the data. LF established the soil mesocosms and performed the experiments, and processed raw DNA sequence data. RD-M contributed to the conception of the work and design of the experiments. LK contributed to the conception of the work and design of the experiments, oversaw the project and helped with data analysis, and manuscript preparation.

### Conflict of Interest

The authors declare that the research was conducted in the absence of any commercial or financial relationships that could be construed as a potential conflict of interest.

## Supplementary Material

Table S1 Analysis of variance assessing the differences in soil chemical and *Streptomyces* community characteristics, bacterial community alpha diversity and richness, and relative abundance of the three most abundant bacterial phyla in low- and high-nutrient soil in response to carbon amendments (^NS^*p* > 0.05; ***p* < 0.05).

Table S2 Soil chemical and *Streptomyces* community characteristics, and bacterial community alpha diversity and OTU richness, and relative abundance of the three most abundant bacterial phyla in carbon-amended and non-amended low- and high-organic matter soils. For each treatment, the mean value and standard error of the mean are reported. F-statistic and *p*-values correspond to the comparison between low and high doses the same carbon substrate (^NS^*p* > 0.05; **p* < 0.1; ***p* < 0.05).

Table S3 Pearson's correlation matrix of soil chemical and *Streptomyces* community characteristics in low- and high-organic matter soils. Values indicate Pearson's correlation coefficient (*r*^2^, top) and *p*-value (bottom) for each comparison.

Table S4 Spearman's rank test of soil chemical, *Streptomyces* community metrics, and dominant bacterial phyla in low- and high-organic matter soils. Values indicate Spearman's Rho values (top) and *p*-values (bottom) corrected for multiple tests using a false discovery rate (FDR) method.

Figure S1 Effect of carbon amendments on intensity of *Streptomyces* inhibition of *Streptomyces scabies-Ss* **(A)** and *Fusarium oxysporum-Fo* **(B)**. Each bar represents the mean percent change from non-amended controls. Positive and negative values indicate the percentage of increase or decrease from control, respectively. Error bars represent ± 1 standard error of the mean.

Figure S2 Bacterial communities in carbon-amended and non-amended soils. Non-metric multidimensional scaling (NMDS) ordination of Hellinger transformed Bray-Curtis dissimilarities of operational taxonomic unit (OTU) counts for low- and high-organic matter soils. Points represent individual samples from mesocosms amended with low (open circles) or high (solid circle) doses of fructose (blue), glucose (orange), malic acid (green), and a mixture of these substrates (pink). Mesocosms amended with sterile water served as experimental controls (black open squares). Significant differences in community composition among carbon amendment dose were assessed using permutational analysis of variance on distance matrices (PERMANOVA). *Adonis R*^2^ and *p*-values are indicated in the lower part of each plot. NMDS stress values were <0.20 in all cases.

Figure S3 Bacterial community compositions in carbon-amended and non-amended soils were significantly correlated with metrics of soil chemistry and *Streptomyces* populations. Non-metric multidimensional scaling (NMDS) ordination of Hellinger transformed Bray-Curtis dissimilarities of OTU counts for low- and high-organic matter soils. Points represent individual samples from mesocosms amended with low (open circles) or high (solid circle) doses of fructose (blue), glucose (orange), malic acid (green), and a mixture of these substrates (pink). Arrows indicate significant correlates of the two NMDS dimensions (NMDS1 and NMDS2) with arrow length indicating correlation strength.

Figure S4 Bacterial community alpha diversity (Shannon H' index, **A**), observed OTU richness **(B)**, and relative abundance of major bacterial phyla **(C)** in carbon amended and non-amended low- and high-organic matter soils. In **(A,B)**, lines inside boxes represent the median, while the whiskers represent ± 1 standard error of the mean.

Figure S5 Effect of carbon amendments on relative abundances of major bacterial order in low- and high-organic matter soils (**A,B**, respectively).
