# Supplementary figures and images for: Carbon Amendments Influence Composition and Functional Capacities of Indigenous Soil Microbiomes
Source: Front Mol Biosci. 2020 Jan 9;6:151. doi: 10.3389/fmolb.2019.00151 (PMC6964746; doi:10.3389/fmolb.2019.00151)

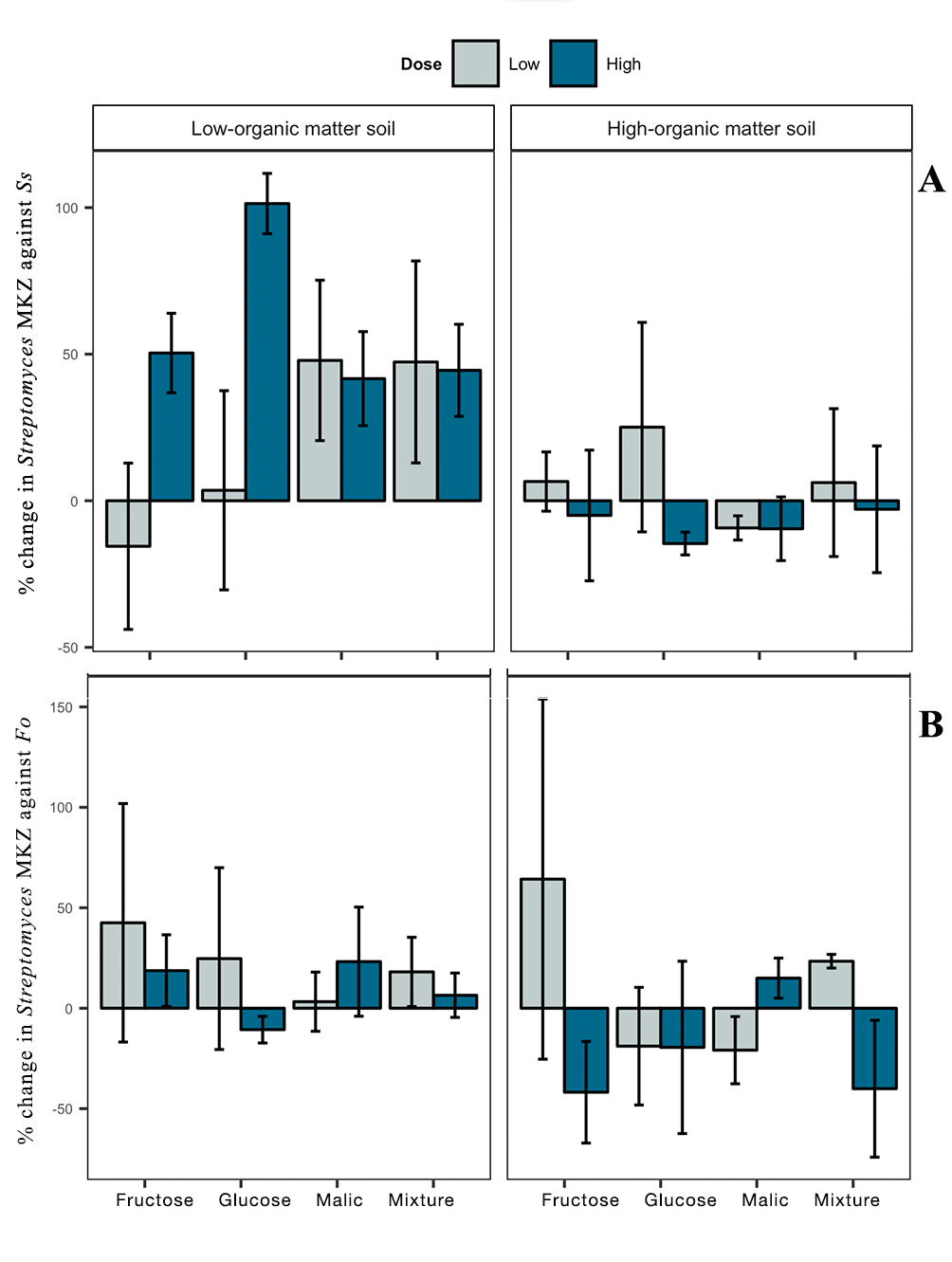

Supplement: Figure S1 — Effect of carbon amendments on intensity of Streptomyces inhibition of Streptomyces scabies-Ss (A) and Fusarium oxysporum-Fo (B). Each bar represents the mean percent change from non-amended controls. Positive and negative values indicate the percentage of increase or decrease from control, respectively. Error bars represent ± 1 standard error of the mean. [file Image_1.tif]

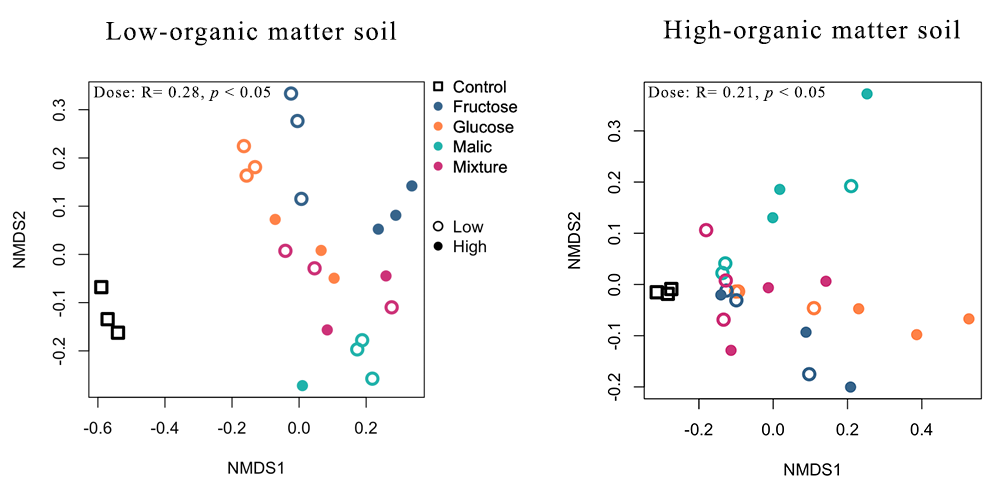

Supplement: Figure S2 — Bacterial communities in carbon-amended and non-amended soils. Non-metric multidimensional scaling (NMDS) ordination of Hellinger transformed Bray-Curtis dissimilarities of operational taxonomic unit (OTU) counts for low- and high-organic matter soils. Points represent individual samples from mesocosms amended with low (open circles) or high (solid circle) doses of fructose (blue), glucose (orange), malic acid (green), and a mixture of these substrates (pink). Mesocosms amended with sterile water served as experimental controls (black open squares). Significant differences in community composition among carbon amendment dose were assessed using permutational analysis of variance on distance matrices (PERMANOVA). Adonis R2 and p-values are indicated in the lower part of each plot. NMDS stress values were <0.20 in all cases. [file Image_2.tif]

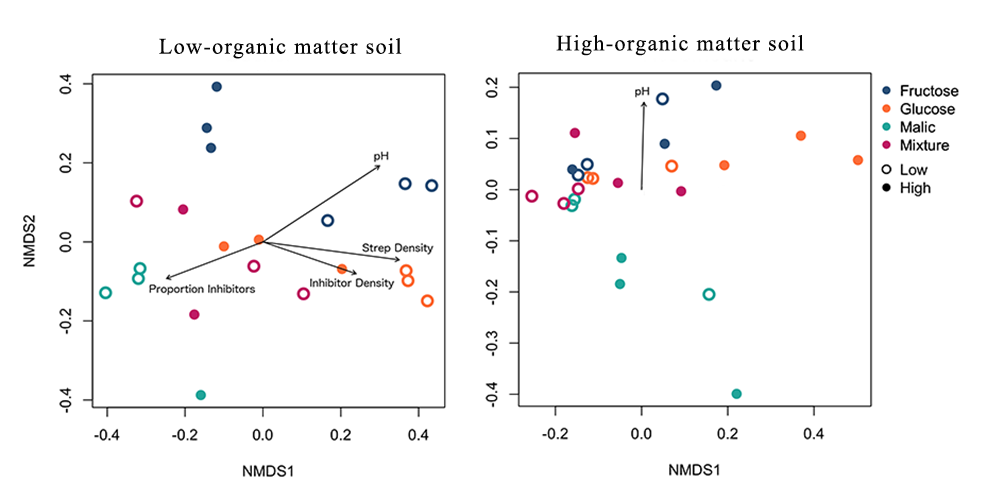

Supplement: Figure S3 — Bacterial community compositions in carbon-amended and non-amended soils were significantly correlated with metrics of soil chemistry and Streptomyces populations. Non-metric multidimensional scaling (NMDS) ordination of Hellinger transformed Bray-Curtis dissimilarities of OTU counts for low- and high-organic matter soils. Points represent individual samples from mesocosms amended with low (open circles) or high (solid circle) doses of fructose (blue), glucose (orange), malic acid (green), and a mixture of these substrates (pink). Arrows indicate significant correlates of the two NMDS dimensions (NMDS1 and NMDS2) with arrow length indicating correlation strength. [file Image_3.tif]

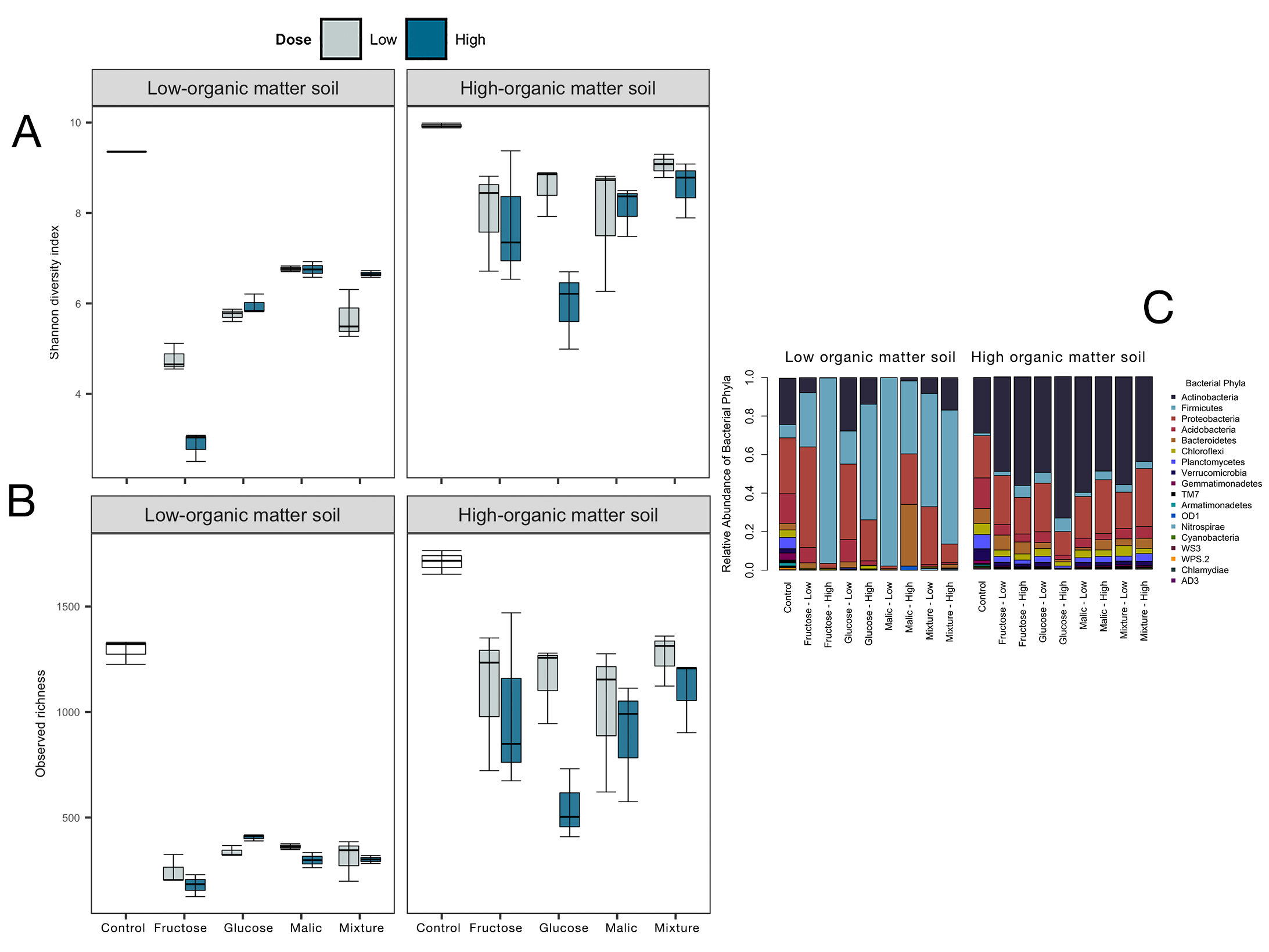

Supplement: Figure S4 — Bacterial community alpha diversity (Shannon H' index, A), observed OTU richness (B), and relative abundance of major bacterial phyla (C) in carbon amended and non-amended low- and high-organic matter soils. In (A,B), lines inside boxes represent the median, while the whiskers represent ± 1 standard error of the mean. [file Image_4.tif]

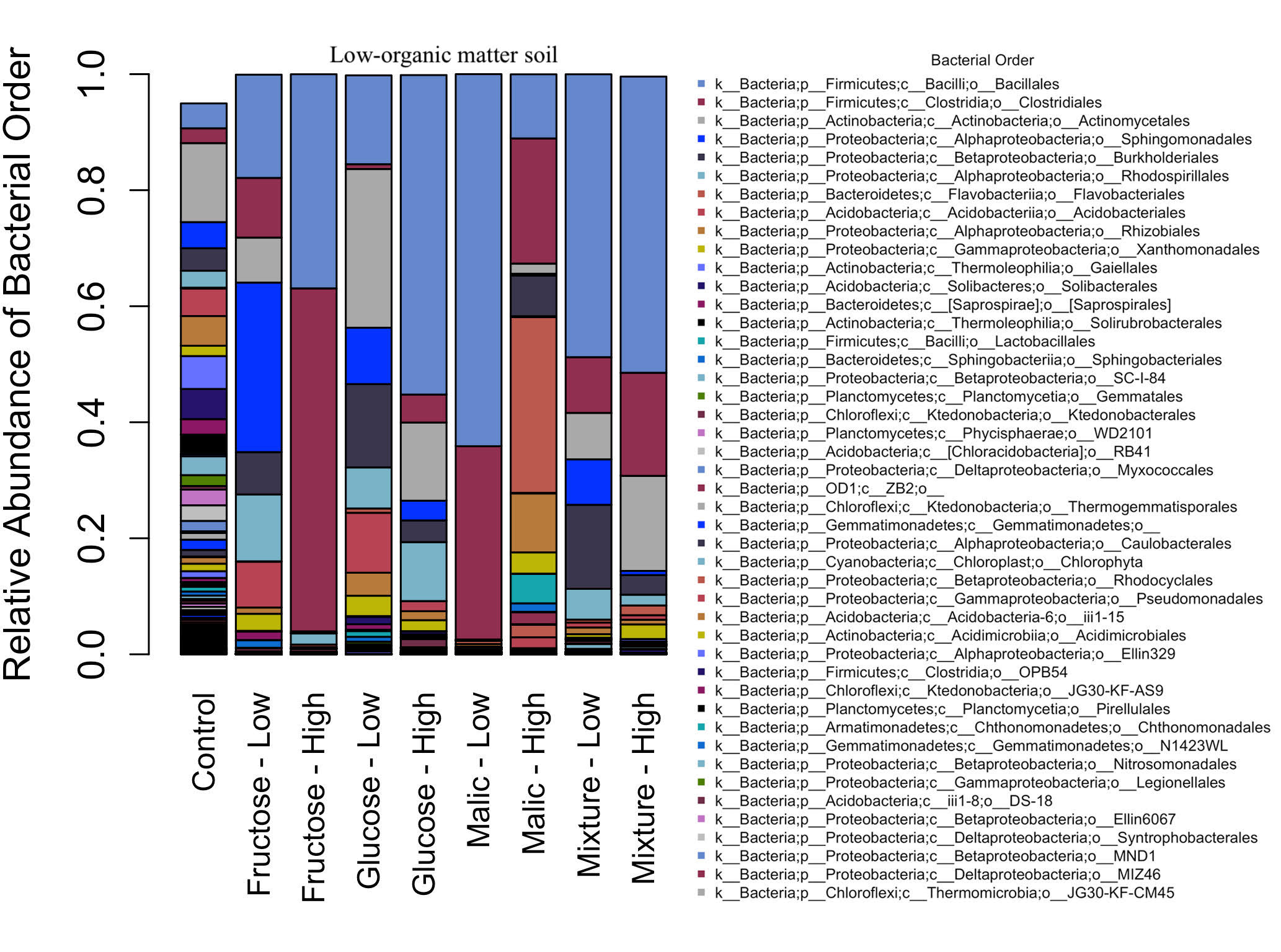

Supplement: Figure S5 — Effect of carbon amendments on relative abundances of major bacterial order in low- and high-organic matter soils (A,B, respectively). [file Image_5.JPEG]

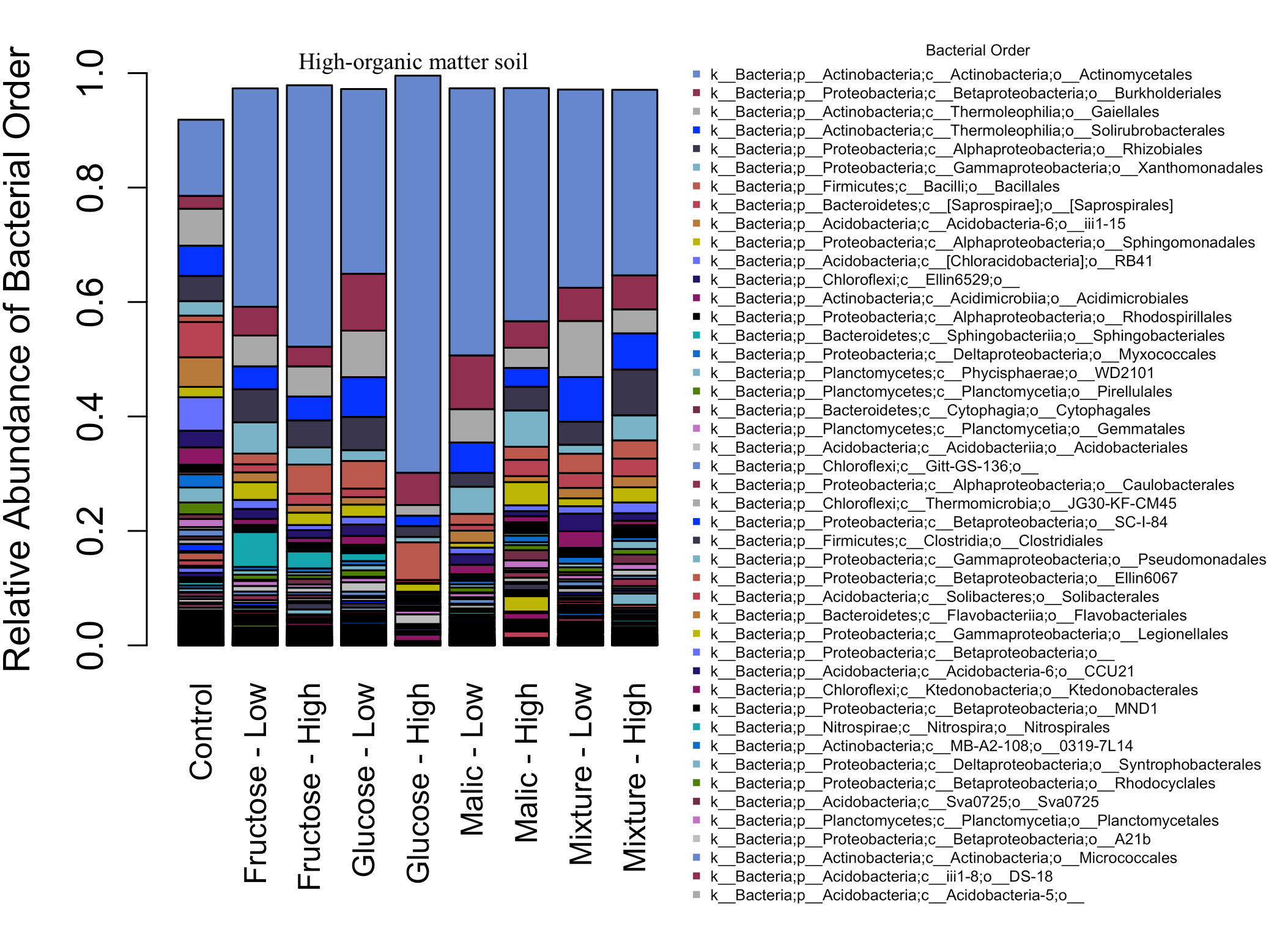

Supplement: Supplementary file 10 [file Image_6.JPEG]
